# Factors Affecting Care-Level Deterioration among Older Adults with Mild and Moderate Disabilities in Japan: Evidence from the Nationally Standardized Survey for Care-Needs Certification

**DOI:** 10.3390/ijerph19053065

**Published:** 2022-03-05

**Authors:** Ai Suzuki, Xueying Jin, Tomoko Ito, Satoru Yoshie, Tatsuro Ishizaki, Katsuya Iijima, Nanako Tamiya

**Affiliations:** 1 Master’s Program in Public Health, Graduate School of Comprehensive Human Sciences, University of Tsukuba, Tsukuba 305-8575, Japan; s2021438@s.tsukuba.ac.jp; 2 Department of Health Services Research, Faculty of Medicine, University of Tsukuba, Tsukuba 305-8577, Japan; tito@md.tsukuba.ac.jp (T.I.); tatsuro@tmig.or.jp (T.I.); ntamiya@md.tsukuba.ac.jp (N.T.); 3 Health Services Research and Development Center, University of Tsukuba, Tsukuba 305-8577, Japan; yoshies-tky@umin.ac.jp; 4 Institute of Gerontology, The University of Tokyo, Bunkyo-ku, Tokyo 113-8656, Japan; iijimakatsuya@gmail.com; 5 Institute for Future Initiatives, The University of Tokyo, Bunkyo-ku, Tokyo 113-0033, Japan; 6 Department of Health Policy and Management, School of Medicine, Keio University, Shinjuku-ku, Tokyo 160-8582, Japan; 7 Human Care Research Team, Tokyo Metropolitan Institute of Gerontology, Itabashi-ku, Tokyo 173-0015, Japan

**Keywords:** care level, instrumental activities of daily living, cognitive function, behavioral problems, survey for care-needs certification

## Abstract

This study aims to investigate the factors of care-level deterioration in older adults with mild and moderate disabilities using nationally standardized survey data for care-needs certification. We enrolled people aged 68 years or older, certified as support levels 1–2 (mild disability) or care levels 1–2 (moderate disability) with no cancer. The outcome was care-level deterioration after two years. The possible factors were physical and mental functions which were categorized as the following five dimensions according to the survey for care-needs certification: body function, daily life function, instrumental activities of daily living (IADL) function, cognitive function, and behavioral problems. A multivariate logistic regression analysis was conducted after stratifying the care level at baseline. A total of 2844 participants were included in our analysis. A low IADL function was significantly associated with a risk of care-level deterioration in all participants. In addition, low cognitive function was linked to care-level deterioration, except for those with support level 1 at baseline. Participants with more behavioral problems were more likely to experience care-level deterioration, except for those with care level 2 at baseline. Our study showed the potential utility of the care-needs certification survey for screening high-risk individuals with care-level deterioration.

## 1. Introduction

In Japan, the proportion of older adults aged 65 years or older was 28.4% in 2020, which is the highest in the world, and it is expected to reach 38.4% in 2065 [1]. To respond to the aging society, the Japanese government implemented the national long-term care insurance (LTCI) system in 2000, aiming to provide suitable care services through care-level assessment. Since then, LTCI services have been provided according to a certificate showing the need for long-term care. There are seven levels of care-need certificates starting with support levels 1 and 2 (mild disability), followed by care levels l and 2 (moderate disability) and care levels 3–5 (severe disability) [2]. Older adults certified as support levels 1 and 2 are able to live independently, but require some assistance with daily life activities, while, those certified as care levels 1 and 2 require more assistance in daily life activities in comparison. Moreover, if older adults are certified as care levels 3 or more, they qualify for admission to a nursing home as they are unable to look after themselves. According to a previous validation study [3], the median Barthel index score by care-need level was as follows: support level 1, 95; support level 2, 90; care level 1, 85; care level 2, 70; care level 3, 60; care level 4, 30; and care level 5, 20. Recently, care-level deterioration has become a critical issue, because it is expected to increase long-term care costs [4] and it involves a low functional status, which increases the risk of mortality [5]. In 2020, the rate of care-level deterioration in a year was highest among those with support level 1 (19.8%), followed by those with care level 1 (17.4%), support level 2 (14.5%), and care level 2 (14.2%) [6]. This highlights the importance of preventing care-level deterioration, by focusing on older adults with mild and moderate disabilities [6].

Previous studies have reported that older age [7,8,9,10,11,12,13] and female sex [8,14] were factors of care-level deterioration, and these associations have been widely recognized. However, the association between care-level deterioration and older adults’ functional status, including physical [15,16,17] and mental functions [8,9,15,17] were inconsistent. One possible reason is that most studies depend on a small sample size [15,18] and a unique questionnaire, which contains different measurements [15,17,18]. Moreover, none of the studies have focused on people with mild or moderate disabilities. 

In Japan, there is a nationally standardized measure of the physical and mental functions of older adults—the survey for care-need certification [19] conducted by the municipalities. Municipalities are responsible for collecting and storing the data for monitoring and improving the quality of care. However, little is known about how to utilize this administrative data for evaluating quality of care, including the prevention of care-level deterioration. This study shows the use of survey data for care-need certification to improve the quality of long-term care. The factors of care-level deterioration can be considered when municipal officers screen individuals at high risk for care-level deterioration. 

In this context, the present study aimed to investigate certain factors, focusing on physical and mental functions that are associated with care-level deterioration among older adults with mild and moderate disabilities using data from the survey for care-need certification.

## 2. Materials and Methods 

### 2.1. Survey for Care-Need Certification

The questionnaire used in the survey for care-need certification consists of 74 items, including 62 items regarding physical and mental functions and 12 items regarding the use of medical procedures [19]. The 62 items are further divided into the following five dimensions (Table 1) [19,20]: (1) Body function, which consists of items such as paralysis and turning over in bed, indicates ability related to physical functions and basic movements; (2) Daily life function, which consists of items such as transferring and eating, indicates functions necessary to maintain life; (3) Instrumental activities of daily living (IADL) function, which consists of items such as taking medicine and shopping for daily necessities, indicates abilities related to IADL; (4) Cognitive function, which consists of items such as remembering own name and short-term memory, indicates the degree of cognitive function; and (5) Behavioral problems, which consists of items such as emotional instability and reversal of day and night, indicates the status of behavioral disorders due to dementia [19]; therefore, we can consider behavioral problems as a proxy of behavioral and psychological symptoms of dementia (BPSD). 

This survey was carried out by trained medical or social specialists, and the results were entered into the computer to calculate the applicant’s standardized score for the five dimensions. Each dimension is allotted a weighted score for each of the component items by statistical methods, to create a total score for each dimension ranging from 0 (completely dependent) to 100 points (completely independent) [19]. Subsequently, the computer assigns the care level by estimating the time for the eight categories of care (eating, toileting, transferring, grooming/bathing, assistance with instrumental activities of daily living, behavioral problems, rehabilitation, and medical services) using scores for the five dimensions and responses to each questionnaire item. The Care Needs Certification Board, comprising physicians, nurses, and other experts in health and social services appointed by municipality, determines whether the initial assessment is appropriate, considering the applicant’s primary care physician’s statement and assessor’s notes during the home visit [19].

Once older adults have been certified, the certified care level is available for a maximum of two years; however, they are allowed to reapply for care-need certification whenever they experience functional changes.

### 2.2. Data Source

We linked the survey data for care-need certification with the medical claims data and basic resident registration data from the municipal government of Kashiwa City, a suburb in the Tokyo metropolitan area. Medical claims data record the types of medical services received and the existing comorbidities when a patient is examined. Basic resident registration data include information on age, sex, date of death, and the reason behind and date of moving in or moving out.

### 2.3. Participants

In the present study, we enrolled those who were (1) certified as support level 1–2 (mild disability) or care level 1–2 (moderate disability) between July 2012 and March 2013, and (2) aged 68 years or older (data were provided as five-year ranges of birth years [e.g., 1945–1950, 1950–1955]). We excluded those who (1) died in the follow-up period, (2) moved to another city during the follow-up period, (3) did not renew care certification in the follow-up period, and (4) were diagnosed with cancer at baseline (i.e., cancer patients are likely to die before functional deterioration occurs; thus, we cannot follow the functional deterioration) [21].

### 2.4. Outcome

The outcome was defined as care-level deterioration during the subsequent two years. The care-level deterioration was calculated by subtracting the care level at baseline from that two years later. If the calculated change in care level was >0, the individual belonged to the “deteriorated” group, and if it was ≤0, indicating a maintained or improved care level, the individual belonged to the “maintained” group.

### 2.5. Exposure

The exposure of interest was the score on the five dimensions of physical and mental functions, evaluated according to the survey for care-need certification at baseline.

### 2.6. Covariates

Age and sex were considered as covariates based on a literature review. We also adjusted for chronic diseases, such as cerebrovascular diseases, cardiac diseases, fractures, and joint diseases. These diseases were selected because they were the dominant causes for long-term care requirements [1]. The ICD-10 codes in the medical claims data, three months prior to the baseline, were used to identify each disease as follows: cerebrovascular diseases were defined as I60–I69; cardiac diseases were defined as I20–I25, I40, I45, and I47–I49; fractures were defined as S27, S02, S12, S22, S32, S42, S52, S62, S82, S92, T02, T08, T02, T08, T10, and T12; and joint diseases were defined as M05, M06, M00–M03, M07, and M10–M25 [22].

### 2.7. Statistical Analysis

First, we compared the distribution of exposure and covariates between the two groups with and without deterioration in care level using chi-square or Student’s *t*-test, after stratifying the baseline care level. Second, we conducted a multivariate logistic regression to examine the association between exposure and care-level deterioration. 

Furthermore, a sensitivity analysis was performed using death and care-level deterioration as a composite outcome. The significance level was set at *p* < 0.05. The statistical software package STATA Version 15 was used in the analysis.

### 2.8. Ethical Approval

This study was approved by the ethics committee of the Faculty of Medicine, University of Tsukuba (approval numbers: 1447-2).

## 3. Results

Of the 4154 participants who met the inclusion criteria, 2844 participants were included in the analysis, after excluding those who died (*n* = 496), moved to another city (*n* = 145), did not renew care-need certification (*n* = 251), or were diagnosed with cancer (*n* = 418).

Table 2 shows the baseline characteristics of participants according to baseline care level. Of the 2844 participants, 720 were categorized as support level 1 (212 male, 508 female), 746 were support level 2 (191 male, 555 female), 749 were care level 1 (239 male, 510 female), and 629 were care level 2 (187 male, 442 female). The proportion of care-level deterioration was 55.7% (401/720), 44.5% (332/746), 42.5% (318/749), and 35.5% (223/629) for support level 1, support level 2, care level 1, and care level 2, respectively.

Table 3 shows the results of multivariate logistic regression analysis. A low IADL function score was significantly associated with a risk of care-level deterioration for all care levels: support level 1 (OR = 1.02, 95% CI = 1.01–1.03), support level 2 (OR = 1.02, 95% CI = 1.01–1.03), care level 1 (OR = 1.01, 95% CI = 1.004–1.02) and care level 2 (OR = 1.02, 95% CI = 1.003–1.03). Moreover, a low cognitive function score was significantly associated with a risk of care-level deterioration except for support level 1; support level 2 (OR = 1.07, 95% CI = 1.02–1.13), care level 1 (OR = 1.02, 95% CI = 1.005–1.04) and care level 2 (OR = 1.03, 95% CI = 1.01–1.05), and a low behavioral problems score was significantly associated with a risk of care-level deterioration except for care level 2; support level 1 (OR = 1.10, 95% CI = 1.02–1.18), support level 2 (OR = 1.04, 95% CI = 1.01–1.08) and care level 1 (OR = 1.01, 95% CI = 1.004–1.02). In the sensitivity analysis, the factors significantly associated with a risk of care-level deterioration and death were consistent with the main analysis.

## 4. Discussion

This study investigated factors associated with care-level deterioration in non-cancer older adults with mild and moderate disabilities using standardized survey data for care-need certification. We found that a low IADL function, low cognitive function, and more behavioral problems were associated with care-level deterioration. To our knowledge, this is the first study that focused on older adults with mild and moderate disabilities using a large sample based on nationally standardized survey data.

We found that a low IADL function was associated with subsequent care-level deterioration for all care levels. Since the study participants had mild and moderate disabilities, we were able to assume that many of them maintained their ADL function but experienced a decreasing IADL function, which was more difficult to perform. Lawton [23] also suggested that there are hierarchical stages of competence, and IADL is more difficult to perform than ADL. Furthermore, previous studies [24,25,26] reported that deterioration of the IADL function preceded the deterioration of ADL. Thus, the IADL function might deteriorate prior to the ADL function, and people with a low IADL function are expected to engage in less activity in their daily lives. This lifestyle might lead to the further deterioration of physical and mental functions, which may ultimately lead to care-level deterioration.

We also found that low cognitive function was associated with subsequent care-level deterioration except for in support level 1. Previous studies [8,15] reported that low cognitive function is associated with care-level deterioration. People with low cognitive function might be less physically active. As a result, in addition so the cognitive function, the physical function deteriorates [27], which may lead to care-level deterioration. However, since older adults with support level 1 have the mildest disability, the degree of cognitive disability was expected to be small, and even if they had a low cognitive function, they could maintain physical activity. Therefore, there was no significant association between low cognitive function and care-level deterioration for those with support level 1. Low cognitive function is not only a risk for care-level deterioration but also a risk factor for mortality and nursing-home utilization [28,29,30]. Thus, cognitive function is an important indicator of functional deterioration.

In addition, more behavioral problems are associated with care-level deterioration except for care level 2. The behavioral problems—included as exposure variables in this study—include items indicating motor hyperactivity, characterized by increased energy levels with more frequent movements [31], such as restlessness and frequent behavior to go out alone. Previous studies [32,33] reported that people with BPSD are at a high risk of falls. Falls might deteriorate physical and mental functions, which in turn lead to care-level deterioration. However, even if older adults with care level 2 have motor hyperactivity, the range and extent of movement would not be large because of the moderate disability in physical and mental functions. Therefore, there was no significant association between behavioral problems and care-level deterioration for those with care level 2.

Some limitations of the present study should be noted. First, this study was conducted in one city, which might lead to problems with generalizability. Second, our covariates are limited to variables that are available in the claims data. Therefore, we were unable to consider covariates such as socioeconomic status and social participation, which were associated with care-level deterioration in previous studies [17]. There might be confounding variables that have not been investigated. Third, the survey data for care-needs certification has not been validated sufficiently except for one study [3]. The reliability of care-needs certification data needs to be verified by combining the other data, such as “Long-term care insurance claims data” or “Medical claims data” in future studies.

Despite these limitations, our findings have practical implications. We demonstrate the importance of assessing IADL function, cognitive function, and behavioral problems as factors of care-level deterioration. Identifying people at risk of care-level deterioration facilitates timely intervention to prevent care-level deterioration. Moreover, we also demonstrated that the survey for care-need certification could be utilized for the assessment of care-level deterioration. This survey data could be used as a tool not only to assess the care level but also to screen those who are at risk of care-level deterioration. The data used in this study are regularly obtained on a nationwide scale, which has the advantage of reducing the burden of data acquisition because existing data can be used. In addition, as the data are obtained by a trained specialist, the reliability of the data is guaranteed. In the future, research needs to be conducted at the national level to verify the generalizability of the obtained results. Furthermore, studies should consider socioeconomic status and social participation as covariates by conducting a primary survey and linking the obtained data with the administrative data.

## 5. Conclusions

In conclusion, our results revealed that a low IADL function, low cognitive function, and more behavioral problems were associated with care-level deterioration after two years in non-cancer older adults with mild and moderate disabilities. Moreover, we found that the survey for care-need certification could be a useful tool for screening those who are likely to experience deterioration in care-level. The results of this study could provide evidence for long-term care-related policymaking and for municipalities in preventing care-level deterioration.

## Figures and Tables

**Table 1:** The five dimensions and their component items.

Dimensions	Items
Body function	Paralysis; Joint contracture; Turning over in bed; Sit up in bed; Sitting; Standing; Walking; Stand up; Single-leg standing; Washing the body; Nail trimming; Eyesight; Hearing ability.
Daily life function	Transferring; Swallowing; Eating; Manage after urination; Manage after defecation; Oral hygiene; Washing face; Combing/Styling hair; Upper-body dressing; Lower-body dressing; Frequency going out.
Instrumental activities of daily living (IADL) function	Taking medicine; Financial management; Daily decision making; Maladaptation to group; Shopping for daily necessities; Cooking.
Cognitive function	Communicate intentions to others; Understanding of daily routine; Remembering own name; Remembering date of birth; Recognizing the season; Location awareness; Short-term memory; Wandering; Being lost.
Behavioral problems	Frequency of feeling persecuted; Making up a story; Emotional instability; Reversal of day and night; Repeating the same story; Shouting; Resisting advice or care; Restlessness; Frequent behavior to go out alone; Collecting items inappropriately; Destruction of things/ clothes; Forgetfulness; Monology; Selfish behavior inappropriate for the situation; Incoherent talk.

**Table 2:** The characteristics of participants at baseline by baseline care-level.

	Support Level 1 *n* = 720			Support Level 2 *n* = 746			Care Level 1 *n* = 749			Care Level 2 *n* = 629		
	Maintained *n* = 319	Deteriorated *n* = 401	*p* Value	Maintained *n* = 414	Deteriorated *n* = 332	*p* Value	Maintained *n* = 431	Deteriorated *n* = 318	*p* Value	Maintained *n* = 406	Deteriorated *n* = 223	*p* Value
Sex *n* (%)												
Male	87 (27.3)	125 (31.2)		94 (22.7)	97 (29.2)		126 (29.2)	113 (35.5)		128 (31.5)	59 (26.5)	
Female	232 (72.3)	276 (68.8)	0.254	320 (77.3)	235 (70.8)	0.043	305 (70.8)	205 (64.5)	0.067	278 (68.5)	164 (73.5)	0.183
Age *n* (%)												
68–72	31 (9.7)	21 (5.2)		50 (12.1)	29 (8.7)		46 (10.7)	31 (9.8)		54 (13.3)	20 (9.0)	
73–77	50 (15.7)	69 (17.2)		73 (17.6)	56 (16.9)		71 (16.5)	47 (14.8)		84 (20.7)	27 (12.1)	
78–82	113 (35.4)	114 (28.4)		130 (31.4)	86 (25.9)		124 (28.8)	86 (27.0)		94 (23.2)	48 (21.5)	
83–87	80 (25.1)	124 (30.9)		106 (25.6)	101 (30.4)		116 (26.9)	94 (29.6)		87 (21.4)	57 (25.6)	
88–	45 (14.1)	73 (18.2)	0.035	55 (13.3)	60 (18.1)	0.010	74 (17.2)	60 (18.9)	0.697	87 (21.4)	71 (31.8)	0.003
Score *n* ± SD												
Body function	91.3 ± 4.3	91.0 ± 4.3	0.335	83.7 ± 6.1	85.0 ± 6.9	0.009	83.7 ± 10.2	85.0 ± 9.5	0.067	73.7 ± 11.2	75.3 ± 12.1	0.097
Daily life function	99.2 ± 1.6	99.0 ± 2.1	0.143	97.7 ± 3.0	97.7 ± 3.0	0.980	94.0 ± 6.2	93.7 ± 6.7	0.518	84.0 ± 8.8	82.5 ± 8.8	0.046
IADL function	85.0 ± 17.6	77.0 ± 20.6	<0.001	80.9 ± 16.6	73.2 ± 19.8	<0.001	55.2 ± 20.0	48.1 ± 18.7	<0.001	44.9 ± 18.0	36.4 ± 16.9	<0.001
Cognitive function	99.4 ± 2.5	98.6 ± 4.3	0.001	99.3 ± 2.4	97.8 ± 5.5	<0.001	94.2 ± 9.1	91.0 ± 11.0	<0.001	92.3 ± 11.7	84.5 ± 18.0	<0.001
Behavioral problems	99.5 ± 1.7	98.7 ± 3.6	0.001	98.3 ± 3.9	96.5 ± 6.9	<0.001	92.9 ± 9.5	89.8 ± 12.3	<0.001	91.2 ± 13.0	88.0 ± 13.8	0.005
Chronic disease *n* (%)												
Cerebrovascular disease	19 (6.0)	38 (9.5)	0.082	48 (11.6)	36 (10.8)	0.747	44 (10.2)	38 (12.0)	0.451	60 (14.8)	22 (9.9)	0.080
Cardiovascular disease	50 (15.7)	52 (13.0)	0.301	62 (15.0)	42 (12.7)	0.362	56 (13.0)	63 (12.3)	0.767	44 (10.8)	24 (10.8)	0.977
Fracture	8 (2.5)	15 (3.7)	0.350	28 (6.8)	11 (3.3)	0.035	28 (6.5)	23 (7.2)	0.693	33 (8.1)	14 (6.3)	0.399
Joint disease	69 (21.6)	73 (18.2)	0.251	98 (23.7)	88 (26.5)	0.374	60 (13.9)	34 (10.7)	0.187	64 (15.8)	25 (11.2)	0.117

SD, standard deviation; IADL; instrumental activities of daily living; Sex, age, and chronic disease compared using chi-square test; Score compared using Student’s *t*-test.

**Table 3:** The factors associated with deterioration: the result of the multivariate logistic analysis.

	Support Level 1 ^a^		Support Level 2 ^a^		Care Level 1 ^a^		Care Level 2 ^a^	
	OR (95% CI)	*p* Value	OR (95% CI)	*p* Value	OR (95% CI)	*p* Value	OR (95% CI)	*p* Value
Body function ^b^	1.04 (0.998–1.08)	0.062	0.99 (0.96–1.01)	0.374	1.004 (0.99–1.02)	0.680	1.01 (0.99–1.03)	0.353
Daily life function ^b^	0.95 (0.86–1.04)	0.274	0.99 (0.93–1.04)	0.586	1.01 (0.98–1.03)	0.649	1.02 (0.998–1.04)	0.074
IADL function ^b^	1.02 (1.01–1.03)	<0.001	1.02 (1.01–1.03)	<0.001	1.01 (1.004–1.02)	0.003	1.02 (1.003–1.03)	0.012
Cognitive function ^b^	1.05 (0.99–1.11)	0.115	1.07 (1.02–1.13)	0.007	1.02 (1.005–1.04)	0.027	1.03 (1.01–1.05)	<0.001
Behavioral problems ^b^	1.10 (1.02–1.18)	0.013	1.04 (1.01–1.08)	0.011	1.01 (1.004–1.02)	0.010	1.001 (0.98–1.02)	0.944

OR, odds ratio; 95% CI, 95% confidential interval; IADL, instrumental activities of daily living; ^a^ Care-level at baseline; ^b^ Per 1-point decrement; Adjusted for age, sex, and chronic disease (cerebrovascular disease, cardiovascular disease, fracture, and joint disease).

## Data Availability

Because this is a secondary study using a municipal government data, data sharing in not applicable.
